# Timing of the first cannulation and survival of arteriovenous grafts in hemodialysis patients: a multicenter retrospective cohort study

**DOI:** 10.1080/0886022X.2021.1988638

**Published:** 2021-10-12

**Authors:** Su-Ju Lin, Shu-Chen Chang, Chun-Wu Tung, Yung-Chien Hsu, Ya-Hsueh Shih, Yi-Ling Wu, Tse-Chih Chou, Chun-Liang Lin

**Affiliations:** a Department of Nephrology, Chang Gung Memorial Hospital, Chiayi, Taiwan; b Research Services Center for Health Information, Chang Gung University, Taoyuan, Taiwan; c Graduate Institute of Clinical Medical Sciences, Chang Gung University, Taoyuan, Taiwan; d Kidney and Diabetic Complications Research Team (KDCRT), Chang Gung Memorial Hospital, Chiayi, Taiwan; e Clinical Informatics and Medical Statistics Research Center, Chang Gung University, Taoyuan, Taiwan; f Kidney Research Center, Chang Gung Memorial Hospital, Taipei, Taiwan; g Center for Shockwave Medicine and Tissue Engineering, Kaohsiung Chang Gung Memorial Hospital, Chang Gung University College of Medicine, Kaohsiung, Taiwan; h College of Medicine, Chang Gung University, Taoyuan, Taiwan

**Keywords:** Arteriovenous graft, survival, hemodialysis, cannulation time

## Abstract

Arteriovenous graft (AVG) is an important vascular access route in hemodialysis patients. The optimal waiting time between AVG creation and the first cannulation is still undetermined, therefore the current study investigated the association between ideal timing for cannulation and AVG survival. This retrospective cohort study used data from the Taiwan National Health Insurance Database, which included 6,493 hemodialysis patients with AVGs between July 1^st^ 2008 and June 30^th^ 2012. The waiting cannulation time was defined as the time from the date of shunt creation to the first successful cannulation. Patients were categorized according to the waiting cannulation time of their AVGs as follows: ≤30 days, between 31 and 90 days, between 91 and 180 days, and >180 days. The primary outcome was functional cumulative survival, measured as the time from the first cannulation to shunt abandonment. The AVGs which were cannulated between 31 and 90 days (reference group) after construction had significantly superior functional cumulative survival compared with those cannulated ≤30 days (adjusted HR = 1.651 with 95% CI 1.482–1.839; *p* < 0.0001) and >180 days (adjusted HR = 1.197 with 95% CI 1.012–1.417; *p* = 0.0363) after construction. An analysis of the hazard ratios in patients with different demographic characteristics, revealed that the functional cumulative survival of AVGs in most groups was better when they received cannulation >30 days after construction. Consequently, in order to achieve the best long-term survival, AVGs should be cannulated at least 1 month after construction, but you should avoid waiting for >3 months.

## Introduction

Hemodialysis is the major therapeutic treatment for patients with end-stage renal disease (ESRD). The Taiwan Renal Registry Data System shows that 88.8% of ESRD patients underwent hemodialysis between 2005 and 2012 [1]. Functional long-term vascular access with sufficient diameter and flow rate is vital for providing efficient hemodialysis. Usually, arteriovenous fistula (AVF) is seen as the preferred vascular access route because it has lower morbidity and mortality rates compared with other access modalities [2–4]. However, arteriovenous graft (AVG) has a wider variety of shapes and configurations for vascular anastomosis, is easier for the surgeon to implant, has a larger surface area available for the initial cannulation, is technically easier to cannulate, and comparatively easy to repair [5–8]. AVG is more suitable for patients with unacceptable arterial or venous anatomy compared with native AVF construction, particularly in elderly or diabetic patients [9]. For this reason, it is just as important to prepare an optimal and functional AVG in the care of hemodialysis patients as it is for AVF.

It is crucial that we investigate variables associated with vascular access maturation and patency. The association between cannulation timing and access survival has been previously researched, but without consistent results [10,11]. When reviewing the current practice guidance worldwide, it was observed that the National Kidney Foundation Dialysis Outcomes Quality Initiative (KDOQI) advised that an AVG can be used almost immediately (early stick AVG) or at 2 weeks (standard AVG) after creation [12]. On the other hand, the Japanese Society of Dialysis Therapy recommended that an AVG should be constructed only 3 to 4 weeks before the initial cannulation [13], and the 2018 Clinical Practice Guidelines by the European Society for Vascular Surgery (ESVS) similarly indorsed that AVGs should be considered for cannulation 2 to 4 weeks after their creation [14]. There is no consensus regarding timing for cannulation. Furthermore, rather than scientific evidence, these current guidelines mainly depend on professional opinions. As a result, in order to determine the ideal cannulating time and its association with AVG survival, we designed a multicenter retrospective cohort analysis of the Taiwan National Health Insurance Research Database (NHIRD).

## Materials and methods

### Data source

The data in our retrospective cohort study was collected from ESRD patients enrolled in the NHIRD. The National Health Insurance (NHI) system, which was initiated in 1995, is a compulsory program in Taiwan and is co-funded by the Taiwanese government, employers, and beneficiaries. All residents, including foreigners, who have lived in Taiwan longer than 6 months are instructed to join the NHI system. The NHIRD contains longitudinal medical records for all health beneficiaries in Taiwan, including patient characteristics, procedures, operations, diagnoses, and fees. The NHIRD and other health-related data, such as cause of death data, were provided by the Health and Welfare Data Science Center (HWDC). Diagnoses in the NHIRD are coded according to the International Classification of Disease, Ninth Revision, Clinical Modification (ICD-9-CM), and records of all interventions also correspond to the relevant codes. Our study had full compliance with the national ethical guidelines, and all data were anonymized before being used for research. Ethical approval was examined and accepted by the Chang Gung Medical Foundation Institutional Review Board (Institutional Review Board number: 104-2195B), and the institutional review board determined that patient consent was not required.

### Study population

All patients with ESRD (ICD-9-CM code: 585) between July 1^st^ 2008 and June 30^th^ 2012 were identified from the NHIRD. The status of ESRD was confirmed by catastrophic illness certification profiles and documented by specialists in the NHI administration. Patients who received AVG (NHI procedure codes: 69032CC, 69034 C) construction were included in the current study. We excluded patients <20 years of age, and patients who had undergone renal transplantation or peritoneal dialysis. Patients who had unclear records regarding shunt construction or cannulation, or who had delayed shunt creation after the initiation of hemodialysis, were also excluded. The selection of study subjects is shown in Figure S1. A total of 6,493 patients with AVG were enrolled in our study, and they were followed until their deaths, deregistration, or the end of our study, whichever came first.

### Outcomes and covariates

For AVG, waiting cannulation time is defined as the time from the date of shunt creation to the date of the first successful cannulation. In our study, we used the initial shunt creation date to minimize mistakes about further revision if patients had several operative records for the shunt. For those patients who never received long-term tunneled catheter insertion before starting hemodialysis, we defined the date of the first hemodialysis as the first successful cannulation. For those who had long-term tunneled catheter insertion, we defined the date of permanent tunneled catheter removal as the first date of successful cannulation because in Taiwan the catheter is usually removed immediately after the first successful cannulation. Moreover, in order to confirm that the cannulation was successful, the included patients needed to have regular hemodialysis records and not have received any intervention (surgical or endovascular) to maintain or restore vascular blood flow within 2 weeks after the defined cannulation date. All kinds of grafts, no matter which anatomical location, materials or operative technique, were included in our analysis.

As explained, there is no consistent opinion across the KDOQI [12], the Japanese Society of Dialysis Therapy [13] or the ESVS [14], regarding whether the waiting cannulation time should be shorter or longer than 4 weeks. KDOQI [12] and ESVS [14] suggest that vascular access should be examined by experienced staff 2 to 6 weeks post-operatively. Therefore, we initially chose a multiple of 30 to define the cannulation waiting time categories, which were grouped as follows: ≤30 days, between 31 and 60 days, between 61 and 90 days and >90 days.

As shown in Figure S2, the AVGs which were cannulated within 30 days of construction had significantly worse functional cumulative survival (adjusted hazard ratio [HR] = 1.693 with 95% confidence interval [CI] 1.486–1.929; *p* < 0.0001). Patients with AVGs which were cannulated ≥91 days after creation had the second worst functional cumulative survival (adjusted HR = 1.18 with 95% CI 1.011–1.378; *p* = 0.0363). No significant difference in the functional cumulative survival was observed between the group where AVGs were cannulated between 31 and 60 days (reference group) and the group where AVGs were cannulated between 61 and 90 days (adjusted HR = 1.067 with 95% CI 0.887–1.285; *p* = 0.4915). In this setting, the functional cumulative survival was similar in AVGs with a waiting cannulation time between 31 and 60 days, and 61 and 90 days. Moreover, the average waiting cannulation time in our subjects was 81.94 days, thus the survival benefits of AVGs waiting for cannulation for ≥91 days were worthy of further consideration. Therefore, the subjects were regrouped by waiting cannulation time as follows: ≤30 days, between 30 and 90 days, between 90 and 180 days, and >180 days, and the following analyses are based on this grouping.

The primary outcome in our study was functional cumulative survival of the AVG, which was defined as the time from the date of first successful cannulation to AVG abandonment. The functional primary patency of an AVG, defined as the time from the date of first successful cannulation to the date of any first intervention (surgical or endovascular) to maintain or restore vascular blood flow, was also investigated. During the observation, censored data included patients who died (1,692, 37.51%), had the lost medical records (2812, 62.34%) and received renal transplantation (7, 0.16%). Covariates included baseline demographic and clinical data, including age, gender, comorbidities, and medications. Comorbidities, such as hypertension (HTN), diabetes mellitus (DM), myocardial infarction (MI), congestive heart failure (CHF), peripheral vascular disease (PVD), and cerebrovascular disease (CVD), were considered when the diagnostic codes occurred in two or more consecutive outpatient records during the 6 months prior to the AVG creation. Medications, including aspirin, clopidogrel, warfarin, and statins, were considered when they were used for at least 1 month, as determined by outpatient records, before or after the AVG construction. In order to clarify the role of cannulation timing, propensity score matching by age, HTN, DM, CHF, CVD, aspirin, clopidogrel, warfarin, and statins was applied.

### Statistical analysis

Data regarding basic demographic characteristics, comorbidities, and medications were presented as the number (percentage) for categorical variables and the mean ± standard deviation (SD). We used Pearson’s chi-squared test and analysis of variance to evaluate the differences in categorical and continuous variables, respectively. The Kaplan-Meier method was used to estimate the functional shunt survival rates. Cox proportional hazard regression models were used to estimate the HRs with 95% CIs. All statistical analyses were conducted using SAS statistical software, version 9.4 (SAS Institute, Inc., Cary, NC, USA).

## Results

**Figure 1.**
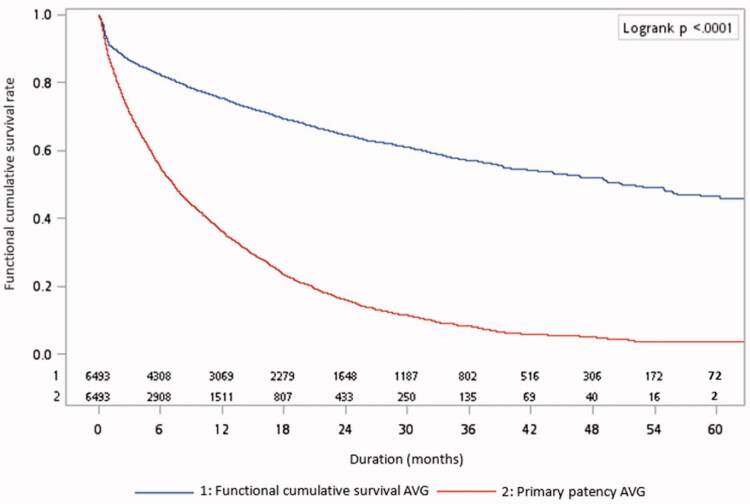
Functional cumulative survival and primary patency of arteriovenous grafts.

Figure 1 displays the survival rates of AVGs. The 1-, 3-, and 5-year functional cumulative survival rates were 75.36%, 57.21%, and 46.48%, respectively. The 1-, 3-, and 5-year functional primary patency rates were 36.19%, 8.22%, and 3.79%, respectively.

### Characteristics of the study subjects

The demographic characteristics, comorbid diseases, and medications of the study subjects are displayed in Table 1. Among the 6,493 patients that participated in this study, 47.9% cannulated their AVGs within 30 days of shunt construction. Patients who cannulated their AVGs within 30 days after their creation were more likely to be male, have CHF comorbidity, and be treated with statins. Patients with AVGs which were cannulated ≥180 days after shunt construction were older, and had a greater probability of having HTN, DM, and CVD, and taking warfarin.

**Table 1.** Characteristics of ESRD patients with AVG (*N* = 6,493).

Demographic characteristic	Waiting cannulation time				*p* Value
	≤30 days	31–90 days	91–180 days	>180 days	
Total patients, n (%)	3110 (47.9)	1889 (29.1)	713 (11.0)	781 (12.0)	
Men, n (%)	1309 (42.1)	792 (41.9)	273 (38.3)	290 (37.1)	0.0269
Age, years, mean ± SD	68.9 ± 12.2	68.1 ± 12.1	68.5 ± 12.0	69.7 ± 12.1	0.0154
Age group, years, n (%)					0.1361
≤30	16 (0.5)	13 (0.7)	4 (0.6)	5 (0.6)	
31–50	216 (6.9)	151 (8.0)	48 (6.7)	54 (6.9)	
51–70	1307 (42.0)	831 (44.0)	312 (43.8)	298 (38.2)	
>70	1571 (50.5)	894 (47.3)	349 (48.9)	424 (54.3)	
Comorbidity, n (%)					
HTN	2107 (67.8)	1165 (61.7)	470 (65.9)	578 (74.0)	<0.0001
DM	1566 (50.4)	850 (45.0)	328 (46.0)	421 (53.9)	<0.0001
MI	124 (4.0)	58 (3.1)	29 (4.1)	23 (2.9)	0.2328
CHF	722 (23.4)	393 (20.8)	131 (18.4)	159 (20.4)	0.0093
PVD	85 (2.7)	48 (2.5)	19 (2.7)	21 (2.7)	0.9824
CVD	404 (13.0)	203 (10.8)	86 (12.1)	115 (14.7)	0.0216
Medication history					
Aspirin	1045 (33.6)	686 (36.3)	248 (34.8)	221 (28.3)	0.0010
Clopidogrel	457 (14.7)	281 (14.9)	107 (15.0)	116 (14.9)	0.9960
Warfarin	89 (2.9)	78 (4.1)	37 (5.2)	43 (5.5)	0.0004
Statins	1059 (34.1)	592 (31.3)	202 (28.3)	158 (20.2)	<0.0001

AVG: arteriovenous graft; CHF: congestive heart failure; CI: confidence intervals; CVD: cerebrovascular disease; DM: diabetes mellitus; HTN: hypertension; MI: myocardial infarction; PVD: peripheral vascular disease.

### First cannulation time and survival of AVGs

**Figure 2.**
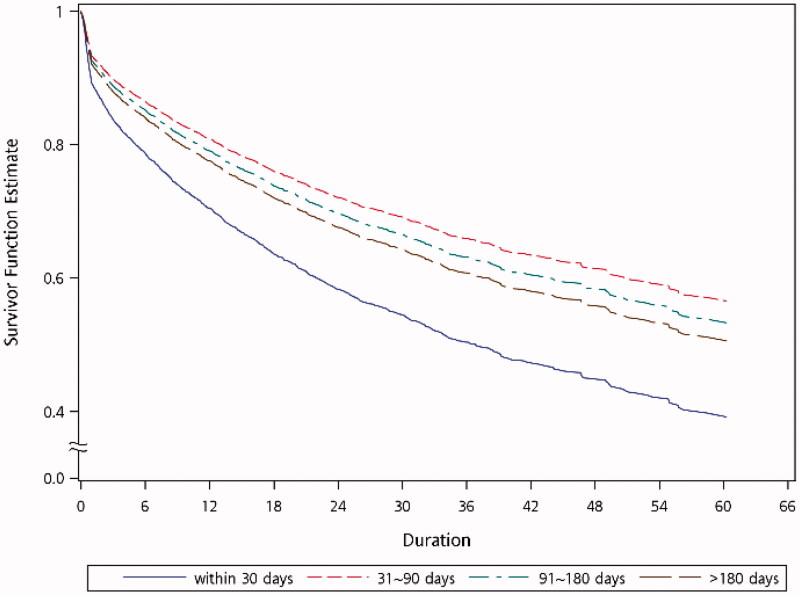
Adjusted Kaplan-Meier plots for functional cumulative survival of grafts. The model was adjusted for age, sex, history of hypertension, diabetes mellitus, myocardial infarction, congestive heart failure, peripheral vascular disease, cerebrovascular disease, and use of aspirin, clopidogrel, warfarin, and statins.

Analysis of the primary outcome, from shunt cannulation to abandonment, is shown in Table 2 and Figure 2. After adjusting for other covariates, patients with a history of DM (adjusted HR = 1.159 with 95% CI 1.043–1.289, *p* = 0.0063) or those who were using warfarin (adjusted HR = 1.266 with 95% CI 1.016 − 1.576, *p* = 0.0354), had significantly inferior functional cumulative survival of their AVGs. AVGs which were cannulated between 31 and 90 days after construction had significantly better functional cumulative survival compared with those being cannulated within 30 days (adjusted HR = 1.651 with 95% CI 1.482–1.839; *p* < 0.0001) or after ≥180 days (adjusted HR = 1.197 with 95% CI 1.012–1.417; *p* = 0.0363).

**Table 2.** Hazard ratios for functional cumulative survival of AVGs.

Characteristic	Event, *n* (%)	Crude HR (95% CI)	*p* Value	Adjusted HR (95% CI)	*p* Value
Waiting cannulation time					
≤30 days	1138 (36.6)	1.650 (1.482–1.838)	<0.0001	1.651 (1.482–1.839)	<0.0001
31–90 days	468 (24.8)	1.000		1.000	
91–180 days	182 (25.5)	1.107 (0.933–1.314)	0.2436	1.105 (0.931–1.312)	0.2528
>180 days	194 (24.8)	1.208 (1.022–1.429)	0.0267	1.197 (1.012–1.417)	0.0363
Age	1982 (30.5)	1.002 (0.999–1.006)	0.2453	1.002 (0.998–1.005)	0.3658
Gender					
Female	1176 (30.7)	1.000		1.000	
Male	806 (30.3)	1.033 (0.944–1.130)	0.4816	1.023 (0.934–1.120)	0.6277
HTN					
No	500 (23.0)	1.000		1.000	
Yes	1482 (34.3)	1.040 (0.938–1.153)	0.4550	0.926 (0.820–1.045)	0.2136
DM					
No	868 (26.1)	1.000		1.000	
Yes	1114 (35.2)	1.115 (1.020–1.219)	0.0169	1.159 (1.043–1.289)	0.0063
MI					
No	1906 (30.5)	1.000		1.000	
Yes	76 (32.5)	1.117 (0.888–1.404)	0.3454	1.102 (0.863–1.407)	0.4378
CHF					
No	1495 (29.4)	1.000		1.000	
Yes	487 (34.5)	1.107 (0.999–1.226)	0.0521	1.086 (0.974–1.212)	0.1371
PVD					
No	1935 (30.6)	1.000		1.000	
Yes	47 (27.2)	0.798 (0.598–1.066)	0.1273	0.755 (0.564–1.011)	0.0589
CVD					
No	1711 (30.1)	1.000		1.000	
Yes	271 (33.5)	1.046 (0.920–1.189)	0.4930	1.016 (0.890–1.159)	0.8144
Aspirin					
No	1711 (30.1)	1.000		1.000	
Yes	271 (33.5)	1.010 (0.921–1.108)	0.8311	1.012 (0.919–1.114)	0.8128
Clopidogrel					
No	1714 (31.0)	1.000		1.000	
Yes	268 (27.9)	0.941 (0.827–1.070)	0.3552	0.927 (0.807–1.063)	0.2770
Warfarin					
No	1898 (30.4)	1.000		1.000	
Yes	84 (34.0)	1.199 (0.964–1.492)	0.1033	1.266 (1.016–1.576)	0.0354
Statins					
No	1367 (30.5)	1.000		1.000	
Yes	615 (30.6)	0.930 (0.845–1.023)	0.1336	0.905 (0.818–1.001)	0.0521

AVF: arteriovenous fistula; AVG: arteriovenous graft; HR: hazard ratio; CHF: congestive heart failure; CI: confidence intervals; CVD: cerebrovascular disease; DM: diabetes mellitus; HTN: hypertension; MI: myocardial infarction; PVD: peripheral vascular disease.

Model was adjusted for age, gender, hypertension, diabetes mellitus, myocardial infarction, congestive heart failure, peripheral vascular disease, cerebrovascular disease, aspirin, clopidogrel, warfarin and statins.

Considering the uneven number of subjects in each group and the divergent associations of several confounders with graft survival, propensity score matching was used for verification and these results are displayed in Table S1 and Table S2. In Table S1, the patients were fully matched by age, HTN, DM, CHF, CVD, aspirin and warfarin. In Table S2, the AVGs which were cannulated within 30 days (adjusted HR = 1.548 with 95% CI 1.262–1.899; *p* < 0.0001) had significantly worse functional cumulative survival compared with the other groups, and the patients with AVGs which were cannulated between 31 and 90 days (reference group) had the lowest HR of functional cumulative survival. This result was consistent with the original grouping.

### Subgroup analysis of cannulation time and survival of AVGs

**Figure 3.**
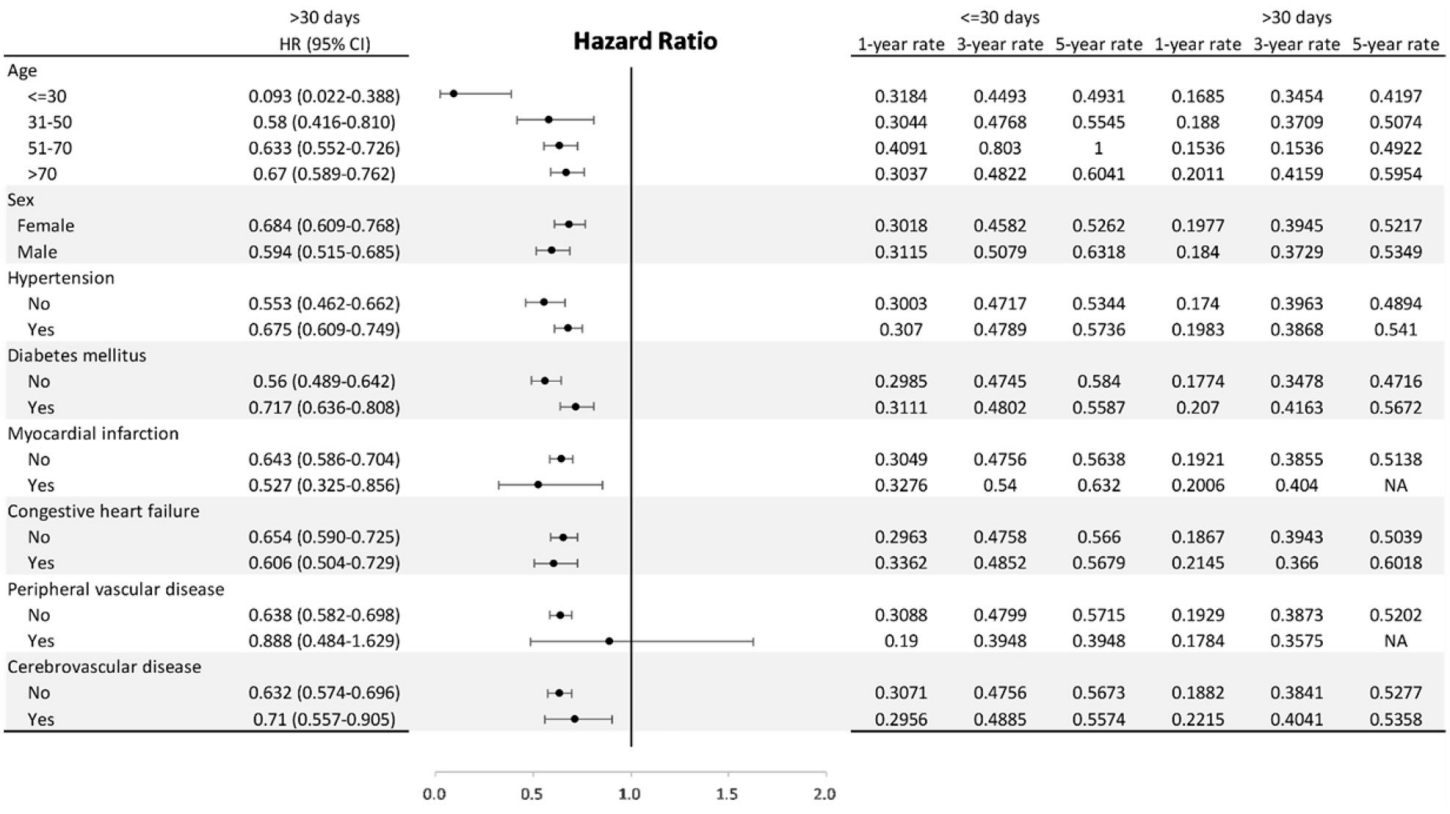
Subgroup hazard ratios for graft survival and AVG creation [<30 days as a reference].

According to the above results, AVGs which were cannulated after ≤30 days had the worst functional cumulative survival. To determine which group of AVGs was more suitable for being cannulated after 30 days, we analyzed the HRs of patients with different demographic characteristics. The forest plots in Figure 3 show that most patients, including groups of different ages, female or male, or those with HTN, DM, MI, CHF or CVD comorbidities, who received AVG cannulation ≥30 days after construction had better functional cumulative survival. The exception was patients with PVD, where there was no significant difference in the functional cumulative survival between needling AVGs within or after 30 days of construction. In addition, we observed that the HRs decreased as the age group decreased. This trend indicates that you should avoid early cannulation of AVGs in younger patients.

## Discussion

In this retrospective cohort study, the analysis was based on a nationwide population database, which represents nearly all people in Taiwan. During a 5-year follow-up period, we analyzed 6,493 incident hemodialysis patients. According to our reliable evidence-based results, AVGs should be cannulated between 31 and 90 days after their creation as this is significantly associated with the highest functional cumulative survival.

Based on clinical needs, there have been some previous small-scale studies with a short-follow-up period, which focused on the association between early cannulation and survival of AVGs. No significant difference was observed in the hematoma, infection and patency rates when cannulation was performed before and after 2 weeks [15,16]. According to the Dialysis Outcomes and Practice Patterns Study (DOPPS) [11], which analyzed 2,730 grafts from hemodialysis patients in seven countries with a diverse 2 to 5-year follow-up period, the relative risk of graft failure was not significantly different between patients with a cannulation time of ≤2 weeks, 2 to 3 weeks, 3 to 4 weeks and >4 weeks, but the relative risks decreased as the waiting cannulation time increased. Moreover, nearly 80% of AVGs in the DOPPS were cannulated before 4 weeks, thus it was still unclear whether AVGs cannulated after 30 days would have more benefits on their survival. On the basis of our larger-population study with a long observative period, AVGs which were cannulated ≤30 days after their creation had the worst functional cumulative survival. In this study, we were looking to determine the perfect time to prepare the pre-dialysis vascular access, for seamless integration with dialysis treatment in chronic kidney disease (CKD) stage 5 patients. According to our results, AVGs which were cannulated between 31 and 90 days had the best functional cumulative survival. Our analysis observed that cannulating AVGs too early (≤30 days) or too late (>90 days) was not beneficial for their survival. The main clinical value of the current study is that we provide important evidence regarding the best timing for puncturing new AVGs. We determined that AVGs should be prepared at least 30 days before the anticipated start date for hemodialysis but ideally not longer than 90 days before.

In our study, the overall 1-year functional cumulative survival of AVGs was 75.36%, and the functional cumulative survival of AVGs which were cannulated between 31 and 90 days after creation was the best. One-year secondary patency rates for AVGs are reported to be between 52 and 85.5% in Japan, where clinicians usually suggest placing AVGs 3 to 4 weeks before the initial cannulation [17–19]. Our functional cumulative survival of AVGs is comparable to the results in Japan, which indicates that cannulating AVGs after 4 weeks is not worse than before 4 weeks. Furthermore, 16% of United States (US) facilities typically cannulated grafts earlier than 2 weeks, and 62% of US facilities cannulated grafts between 2 and 4 weeks in the DOPPS. The estimated 1-year survival probability was 49% for grafts (95% CI 42 to 57%, *N* = 251) in the US group, which is inferior to our 1-year cumulative survival [11,20]. This implies that cannulating AVGs after 4 weeks is better than before 4 weeks. On the other hand, KDOQI guidance recommends creating AVGs 3 to 6 weeks before the initiation of hemodialysis [12]. There is currently no corresponding large-scale study that supports this guidance, however our results support their suggestions in a way. Consequently, according to our evidence-based results, AVGs should be cannulated at least one month after their creation. It should be noted that AVGs in younger patients should not be cannulated too early to obtain better functional cumulative survival, as shown in Figure 3.

The 1-year functional primary patency rates were 36.19% in the current study. These results are somewhat lower than those reported in Japan (35.3–64.5%) [17–19]. When comparing the 1-year functional cumulative survival, our results (75.36%) are better than in the US (49%) [11,20], and are not inferior to those in Japan (52–85.5%) [17–19]. This indicates that more aggressive interventions are performed during access surveillance in Taiwan. The medical convenience and economy of the NHI system may be a major reason for this. There is not much available data to compare the 3-year functional primary patency. Further studies are needed to determine why the patency rate is so low; they should focus on the frequency and duration of each stenosis or thrombosis.

According to our study, the functional cumulative survival of AVGs was similar in patients with and without PVD. The Forest plots demonstrated that the survival of AVGs in patients with PVD was not associated with the needling time before or after 30 days. This means that the underlying vasculature determines the cannulation and survival of AVGs in patients with PVD. Previous studies also revealed that peripheral vessel disease was associated with maturation and failure, and a decreased patency of vascular access [21,22]. Compared with AVFs where the flow increases over time, the cannulation time for AVGs depends on the period allowed for healing, the grafts incorporation into the tissue and the resolution of local tissue swelling; failure of AVGs is associated with vessel manipulation, neointimal hyperplasia, inward remodeling and repeat cannulation injury [5,23–25]. For patients with PVD, atherosclerosis of peripheral arteries may affect the inflow arteries of vascular access at the same time, and the functional cumulative survival of AVGs shows no significant difference between early and late cannulation because of their poor vasculature. Consequently, native vascular status plays a key role in the use and failure of AVGs. Considering the best functional cumulative survival of AVGs were those cannulated between 31 and 90 days, the cannulation of AVGs should be performed at least one month after their creation but not after >3 months.

It is worth noting that in our study, 781 patients (12%) used their AVGs >180 days after construction. While the AVGs are waiting to be used, neointimal hyperplasia at the graft-venous junction may develop and cause the AVG dysfunction. On the other hand, this group had a higher proportion of elderly, HTN, DM and CVD patients. The vasculature in this group will be worse and is more likely to associated with atherosclerosis or arterial calcification. More time is needed and higher difficulty is expected for maturation of the pre-dialysis vascular access. Rather than AVF, AVG may be more suitable for these patients. One recent prospective cohort study in Korea observed prevalence of DM, CVD, and PVD were significantly higher in AVG group than AVF and central venous catheter group [26]. This is similar to our suppose. Besides, on the basis of previous studies, we know that elderly, poor blood pressure control, DM and CVD are risk factors for the progression of CKD [27,28]. We usually expected rapid decline of renal functions in these patients. AVGs are created earlier than usual in these high-risk patients, and the timing of the start point for dialysis was sometimes over-estimated. Ultimately, these AVGs waited for cannulation for >180 days. How to predict the start point for dialysis and referral to construct vascular access more accurately is an important and challenging issue for nephrologists.

Our study had a sufficient follow-up period and is based on an analysis of the NHIRD, which covers most of the population of Taiwan. The study population in our study was confirmed by catastrophic illness certification. This therefore lowered the chance of possible mistakes with the diagnostic ICD-9-CM codes, precludes conditions of unexpected shunt cannulation due to acute kidney injury, and indicates almost all hemodialysis patients. AVG creation, each vascular intervention and AVG abandonment were all established using consistent operative and therapeutic codes; we could therefore accurately analyze the first cannulation time and its survival. The results in our study are reliable and an accurate reflection of AVG survival in Taiwan.

However, the current study did have some limitations. First, we could not investigate the laboratory data or clinical parameters in the NHIRD, such as the anatomical location of the graft vein anastomosis, the AVG material, the operative technique for graft creation, preexisting long-term tunneled catheter ipsilateral or contralateral to the AVG, physical status, and blood pressure. Second, we could not assess/understand the indication for the choice of graft implantation, which is usually dependent on an evaluation by the cardiovascular surgeon before or during the operation. Third, the needling process, including the types of needles used and the way the needles were placed, could not be determined by relevant codes for materials or procedure. Fourth, the statistics regarding comorbidities and medications are determined using ICD-9-CM codes following claims for reimbursement, which may have been misclassified.

## Conclusions

According to this multicenter retrospective cohort study, the cannulation timing of shunts has a profound impact on their survival. AVGs that were cannulated between 31 and 90 days after their creation had favorable functional cumulative survival, and our evidence-based results support the KDOQI guidance [5] to some extent. However, further randomized controlled studies should be performed prospectively to verify our findings. We believe that timely construction of AVGs with adequate waiting time and avoiding cannulating AVGs too early, will improve the care of hemodialysis patients.

## Supplementary Material

Supplemental Material
