# Supplementary material for: Timing of the first cannulation and survival of arteriovenous grafts in hemodialysis patients: a multicenter retrospective cohort study
Source: Ren Fail. 2021 Oct 12;43(1):1416–24. doi: 10.1080/0886022X.2021.1988638 (PMC8519537; doi:10.1080/0886022X.2021.1988638)
Supplement: Supplemental Material [file IRNF_A_1988638_SM6442.pdf]

Supplementary Files

Timing of the First Cannulation and Survival of Arteriovenous Grafts in Hemodialysis Patients: A Multicenter Retrospective Cohort Study

Su-Ju Lin, Shu-Chen Chang, Chun-Wu Tung, Yung-Chien Hsu, Ya-Hsueh Shih, Yi-Ling Wu, Tse-Chih Chou, & Chun-Liang Lin

Supplementary Figure S1: Flow chart of patient enrollment from the National Health Insurance Research Database (NHIRD).

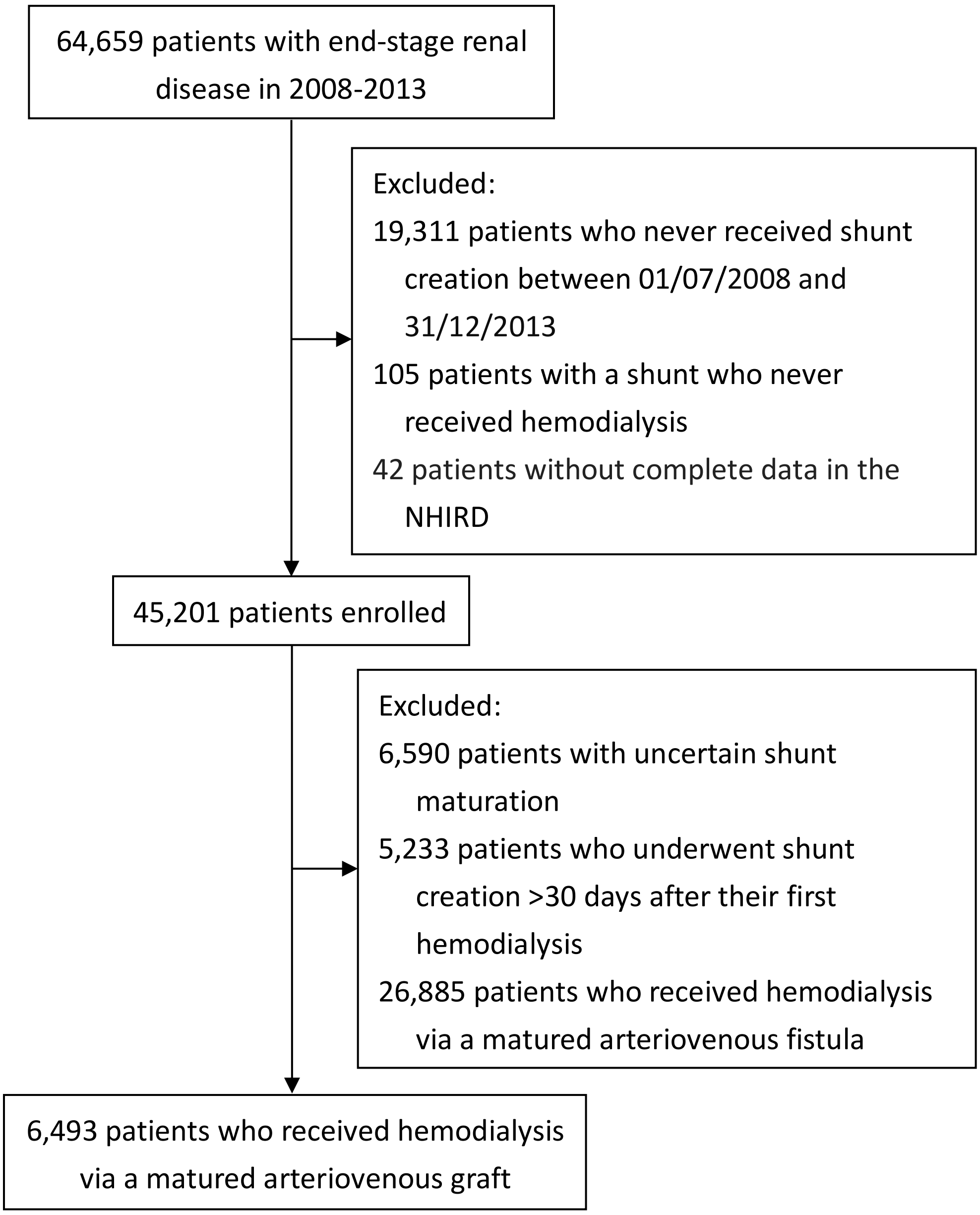

Supplementary Figure S2: Hazard ratios for functional cumulative survival of the AVG grouped by month (N=6,493)

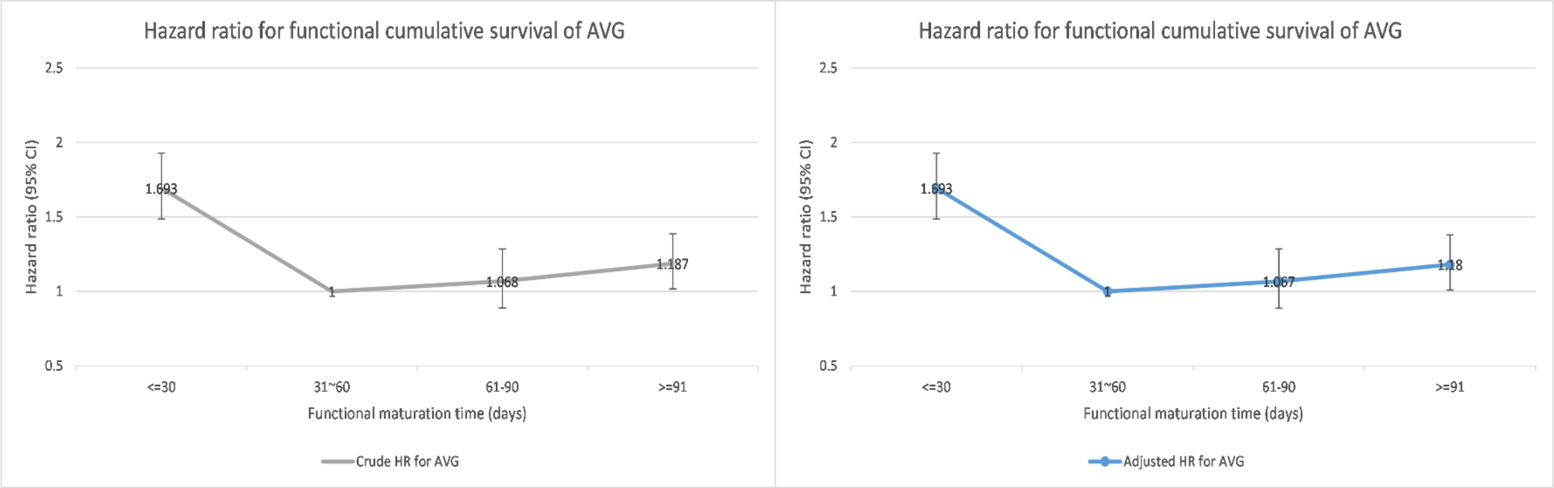

AVG, arteriovenous graft

The model was adjusted for age, gender, hypertension, diabetes mellitus, myocardial infarction, congestive heart failure, peripheral vascular disease, cerebrovascular disease, and use of aspirin, clopidogrel, warfarin, and statins.

Supplementary Table S1: Characteristics of ESRD patients with AVG grouped by propensity score

Demographic characteristic	Waiting puncture time				*P* value
	≤30 days	31-90 days	91-180 days	>180 days	
Total population, n (%)	651 (25)	651 (25)	651 (25)	651 (25)	
Men, n (%)	222 (34.1)	239 (36.71)	245 (37.63)	248 (38.1)	0.4436
Age, years, mean ±SD	69.5 ± 11.5	69 ± 12.2	68.9 ± 12	69.1 ± 12.2	0.7913
Age group, years, n (%)					0.6905
<50	41 (6.29)	54 (8.3)	44 (6.76)	53 (8.14)	
51-70	262 (40.25)	265 (40.71)	281 (43.16)	259 (39.78)	
>70	348 (53.46)	332 (51)	326 (50.08)	339 (52.07)	
Comorbidity, n (%)					
HTN	441 (67.74)	446 (68.51)	456 (70.05)	455 (69.89)	0.7702
DM	306 (47)	303 (46.54)	318 (48.85)	319 (49)	0.7441
MI	27 (4.15)	29 (4.45)	21 (3.23)	22 (3.38)	0.5978
CHF	115 (17.67)	135 (20.74)	126 (19.35)	120 (18.43)	0.5297
PVD	19 (2.92)	20 (3.07)	18 (2.76)	15 (2.3)	0.8495
CVD	66 (10.14)	69 (10.6)	85 (13.06)	80 (12.29)	0.3022
Medication history					
Aspirin	191 (29.34)	216 (33.18)	207 (31.8)	195 (29.95)	0.423
Clopidogrel	86 (13.21)	89 (13.67)	90 (13.82)	90 (13.82)	0.9866
Warfarin	33 (5.07)	33 (5.07)	35 (5.38)	35 (5.38)	0.9888
Statins	167 (25.65)	162 (24.88)	151 (23.2)	155 (23.81)	0.7357

Abbreviations: AVG, arteriovenous graft; CHF, congestive heart failure; CI, confidence intervals; CVD, cerebrovascular disease; DM, diabetes mellitus; HTN, hypertension; MI, myocardial infarction; PVD, peripheral vascular disease

Supplementary Table S2: Hazard ratios for functional cumulative survival of AVG grouped by propensity score

Waiting puncture time	Event, n (%)	Crude HR (95% CI)	*P* value	Adjusted HR (95% CI)	*P* value
≤30 days	218 (33.49)	1.527 (1.247-1.871)	<.0001	1.548 (1.262-1.899)	<.0001
31-90 days	163 (25.04)	1.0000		1.0000	
91-180 days	173 (26.57)	1.167 (0.942-1.445)	0.1585	1.168 (0.942-1.447)	0.1567
>180 days	152 (23.35)	1.153 (0.924-1.439)	0.2081	1.164 (0.932-1.453)	0.1811

Abbreviations: AVG, arteriovenous graft; HR, hazard ratio; CHF, congestive heart failure; CI, confidence intervals; CVD, cerebrovascular disease; DM, diabetes mellitus; HTN, hypertension; MI, myocardial infarction; PVD, peripheral vascular disease

Model was adjusted for age, gender, hypertension, diabetes mellitus, myocardial infarction, congestive heart failure, peripheral vascular disease, cerebrovascular disease, and use of aspirin, clopidogrel, warfarin, and statins
